# Advances in Tumor‐Derived Exosomal Non‐Coding RNAs Regulating M2 Macrophage Polarization: Molecular Mechanisms and Signaling Pathway

**DOI:** 10.1002/cam4.71421

**Published:** 2025-12-08

**Authors:** Yifan Bian, Jilei Li, Jiarui Cao, Sizhe Wang, Chunzheng Ma

**Affiliations:** ^1^ Henan University of Chinese Medicine Zhengzhou Henan China; ^2^ Henan Province Hospital of TCM Zhengzhou Henan China

**Keywords:** exosomes, immune cells, immunosuppressive tumor microenvironment, immunotherapy resistance, M2 macrophage polarization, non‐coding RNA

## Abstract

**Background:**

Cancer persists as a leading cause of global mortality, largely due to the immunosuppressive tumor microenvironment (TME) that facilitates tumor progression and therapy resistance. M2 macrophages dominate this immunosuppressive landscape, and emerging evidence highlights tumor‐derived exosomes (TEXs) as critical mediators of macrophage M2 polarization via delivery of noncoding RNAs (ncRNAs), including miRNAs, lncRNAs, and circRNAs. These TEX‐ncRNA networks activate key signaling pathways (e.g., JAK/STAT, PI3K/AKT, NF‐κB) to sustain immunosuppression and pro‐tumorigenic responses. Understanding the molecular intricacies of TEX‐driven M2 polarization is essential for advancing immunotherapeutic strategies.

**Methods:**

This review systematically analyzes literature (2019–2024, from PubMed and Web of Science) on the molecular mechanisms by which TEX‐derived ncRNAs drive M2 polarization and their interplay with immunotherapies.

**Results and Conclusion:**

This review contains 142 citations, 60 of which are detailed examples of this mechanism. Our analysis of the literature shows that TEXs deliver specific ncRNAs to macrophages, reprogramming them toward an M2 phenotype via pathways such as PTEN/PI3Kγ, Wnt/β‐catenin, and STAT3. This polarization amplifies immunosuppressive factor secretion and promotes tumor metastasis, chemoresistance, and immune evasion. These insights provide a theoretical foundation for novel TME‐targeted therapies, potentially improving outcomes in refractory cancers.

## 1 Introduction

Exosomes (EXOs), nanoscale extracellular vesicles (30–150 nm in diameter), serve as critical mediators of intercellular communication within the TME by transporting bioactive molecules such as proteins, lipids, and nucleic acids [1]. TEX‐RNAs can locally regulate or systemically reshape the biological properties of the TME through circulatory dissemination. Recent research has focused on exosomal RNA components, including mRNAs and non‐coding RNAs (ncRNAs). These functional RNA molecules, upon uptake by recipient cells, influence tumor development and immune microenvironment establishment by inducing metabolic reprogramming, mediating immune suppression, and maintaining tumor heterogeneity [2, 3, 4, 5].

## 2 Role of M2 Macrophage in Tumor Progression

Tumor‐associated macrophages (TAMs), constituting 30%–50% of the tumor immune microenvironment, drive tumor progression through the STAT6/PPARγ axis‐mediated polarization into the M2 phenotype. Their immunosuppressive mechanisms include molecular‐level secretion of IL‐10 and TGF‐β to suppress dendritic cell maturation and recruitment of regulatory T cells (Tregs) via CCL18/CCL22, establishing an immunosuppressive network [6, 7, 8]; metabolic‐level depletion of L‐arginine by arginase 1 (Arg1), leading to T cell dysfunction [6, 9]; and immune checkpoint‐level NF‐κB‐mediated upregulation of PD‐L1 and CD47, inducing CD8+ T cell exhaustion and NK cell inhibition [10, 11, 12, 13]. This multidimensional regulation promotes angiogenesis and epithelial‐mesenchymal transition (EMT) through VEGF/MMP9 secretion, which correlates significantly with clinical therapy resistance [14, 15].

**FIGURE 1:**
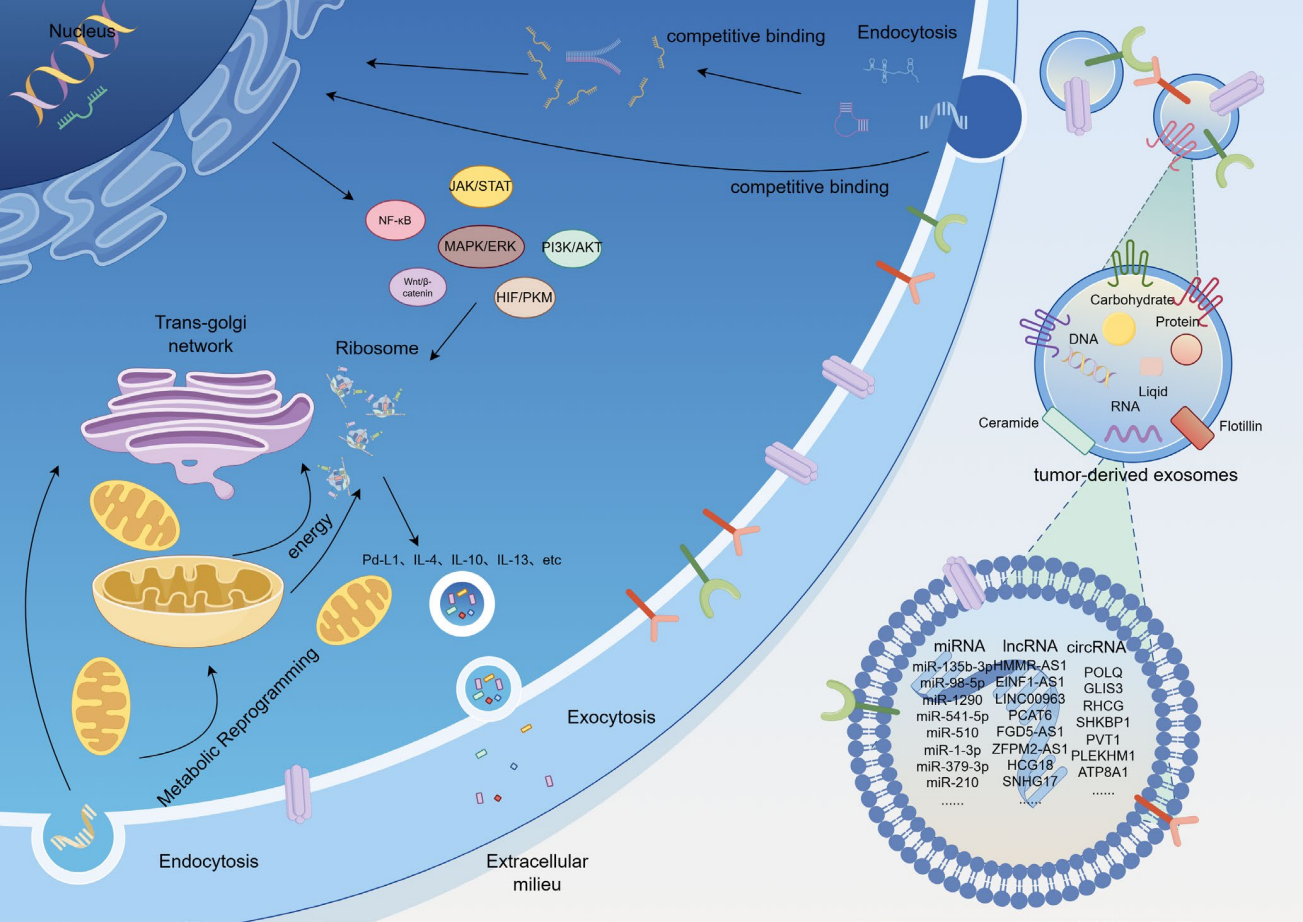
This comprehensive schematic summarizes experimentally validated mechanisms by which exosomal ncRNAs (miRNAs, lncRNAs, and circRNAs) promote macrophage M2 polarization. These exosomes achieve targeted delivery to recipient cells by binding to specific glycoproteins on the macrophage membrane. Upon delivery, the ncRNAs regulate M2 polarization through various mechanisms, including ceRNA networks, nuclear translocation, or direct modulation of key signaling pathways (e.g., JAK/STAT, PI3K/AKT, NF‐κB, MAPK/ERK, HIF‐1α/PKM2, and Wnt/β‐catenin). The activated signaling pathways, in conjunction with some ncRNAs that directly interact with ribosomes or enter mitochondria, collectively promote metabolic reprogramming within the cells, enhance energy production, and facilitate the expression and release of M2‐associated markers (such as PD‐L1, IL‐4, and IL‐13). Ultimately, this process drives macrophage M2 polarization and fosters the formation of an immunosuppressive microenvironment.

While current studies have begun to delineate the immunosuppressive remodeling by TEXs, critical gaps persist in understanding how exosomal nucleic acids orchestrate TAM polarization. Two fundamental questions remain unresolved: (1) the transmembrane delivery mechanisms of exosomal cargo (e.g., non‐coding RNAs) and their intracellular signaling activation cascades, and (2) the dynamic crosstalk between exosomal components and polarization‐related pathways (e.g., JAK/STAT, PI3K/AKT) (Figure 1). Elucidating these mechanisms will advance the molecular decoding of tumor immune editing and establish a theoretical framework for developing exosome‐nucleic acid‐based precision therapeutics targeting TAM reprogramming.

## 3 Interplay Between TEX‐Derived ncRNAs and Macrophage Polarization

**FIGURE 2:**
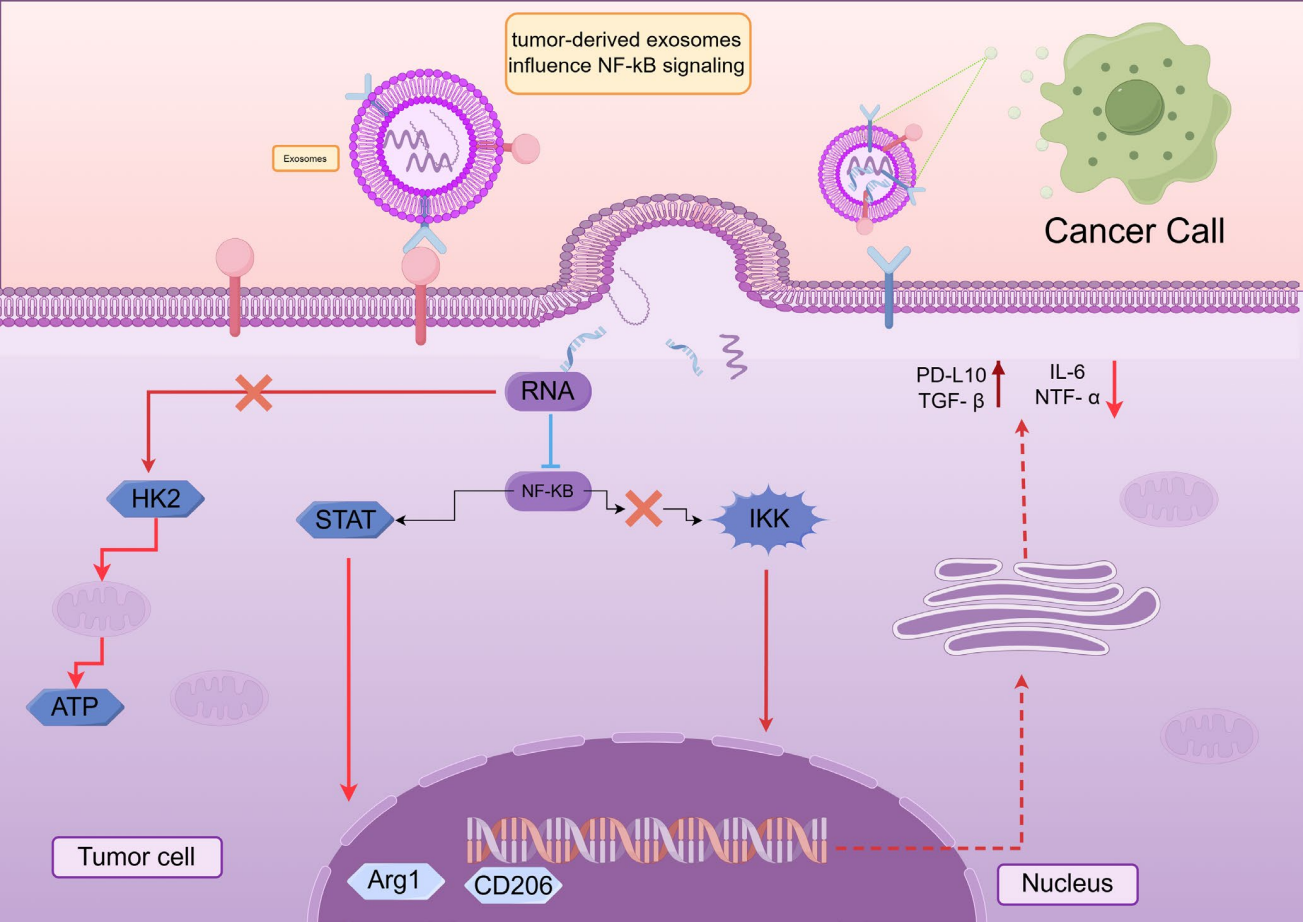
This diagram illustrates the dual regulatory mechanisms by which tumor‐derived exosomal ncRNAs promote macrophage M2 polarization through the NF‐κB signaling pathway. After entering macrophages, these ncRNAs suppress HK2 expression (indicated by a red “X” symbol) to enhance oxidative phosphorylation, thereby providing energy for M2 polarization. (Note: HK2 typically inhibits oxidative phosphorylation in mitochondria, ultimately suppressing energy supply.) Concurrently, they synergistically activate the NF‐κB and STAT pathways to upregulate (represented by red dashed lines, denoting enhanced gene expression) M2‐associated markers (e.g., Arg1 and CD206) and immunosuppressive factors (e.g., PD‐L1 and TGF‐β), while inhibiting IKK‐mediated production of pro‐inflammatory cytokines (such as IL‐6 and TNF‐α). This coordinated regulation ultimately drives M2 polarization.

Recent studies have confirmed that TEX‐derived ncRNAs regulate macrophage polarization by suppressing key regulatory factors (e.g., SOCS3, PTEN) through competing endogenous RNA (ceRNA) interactions and epigenetic modulation, thereby activating critical signaling pathways within macrophages. This dual mechanism induces metabolic reprogramming events such as enhanced glycolysis and fatty acid oxidation, driving the formation of M2‐polarized phenotypes [16, 17, 18, 19] (Figure 2). Furthermore, this regulatory process accelerates the establishment of an immunosuppressive microenvironment through upregulation of immune checkpoint molecules like PD‐L1, which is strongly associated with reduced clinical response rates to PD‐1/CTLA‐4 inhibitors. The underlying mechanisms involve the induction of T cell exhaustion and systemic suppression of the IFN‐γ signaling pathway [10, 11, 20]. Consequently, specific signaling pathways or key regulatory factors governing macrophage M2 polarization represent critical targets, with the following pathways being the primary focus of ongoing research. These regulatory effects are predominantly mediated through the activation of key signaling pathways within macrophages. Among these, certain specific signaling pathways (e.g., JAK/STAT) serve as a central hub and will be discussed in the following section.

## 4 JAK/STAT Signaling Pathway

The JAK/STAT signaling pathway (Janus Kinase/Signal Transducer and Activator of Transcription Pathway), a central hub for extracellular signal transduction, regulates macrophage M2 polarization through phosphorylation cascades [21]. Upon binding of cytokines such as IL‐4/IL‐13 to macrophage surface receptors (e.g., IL‐4Rα), receptor dimerization triggers activation of JAK1/JAK3 kinases, leading to phosphorylation of STAT6 at tyrosine residue Tyr641. This induces STAT6 homodimer formation, nuclear translocation, and direct binding to promoter regions of M2 marker genes (e.g., Arg1, CD206) [22, 23, 24, 25]. The TME establishes a multidimensional regulatory network via TEX‐derived ncRNAs: ceRNA mechanisms (e.g., lncRNA NEAT1 and miR‐125a) sustain JAK activation by stabilizing MINK1 kinase [26, 27]; epigenetic modulation of SOCS3 expression relieves STAT3 autoinhibitory feedback [28]; and enhanced OSMR translation efficiency promotes JAK2/STAT3 axis activation [29]. Notably, STAT6 activation positively upregulates IL‐4Rα expression, while SOCS1‐mediated STAT3 dephosphorylation via STAT5b establishes dynamic equilibrium. This bidirectional regulation enables the pathway to rapidly initiate M2 polarization in response to microenvironmental signals while preventing hyperactivation through negative feedback mechanisms [30, 31].

### 4.1 ceRNA‐Dependent Regulation

**FIGURE 3:**
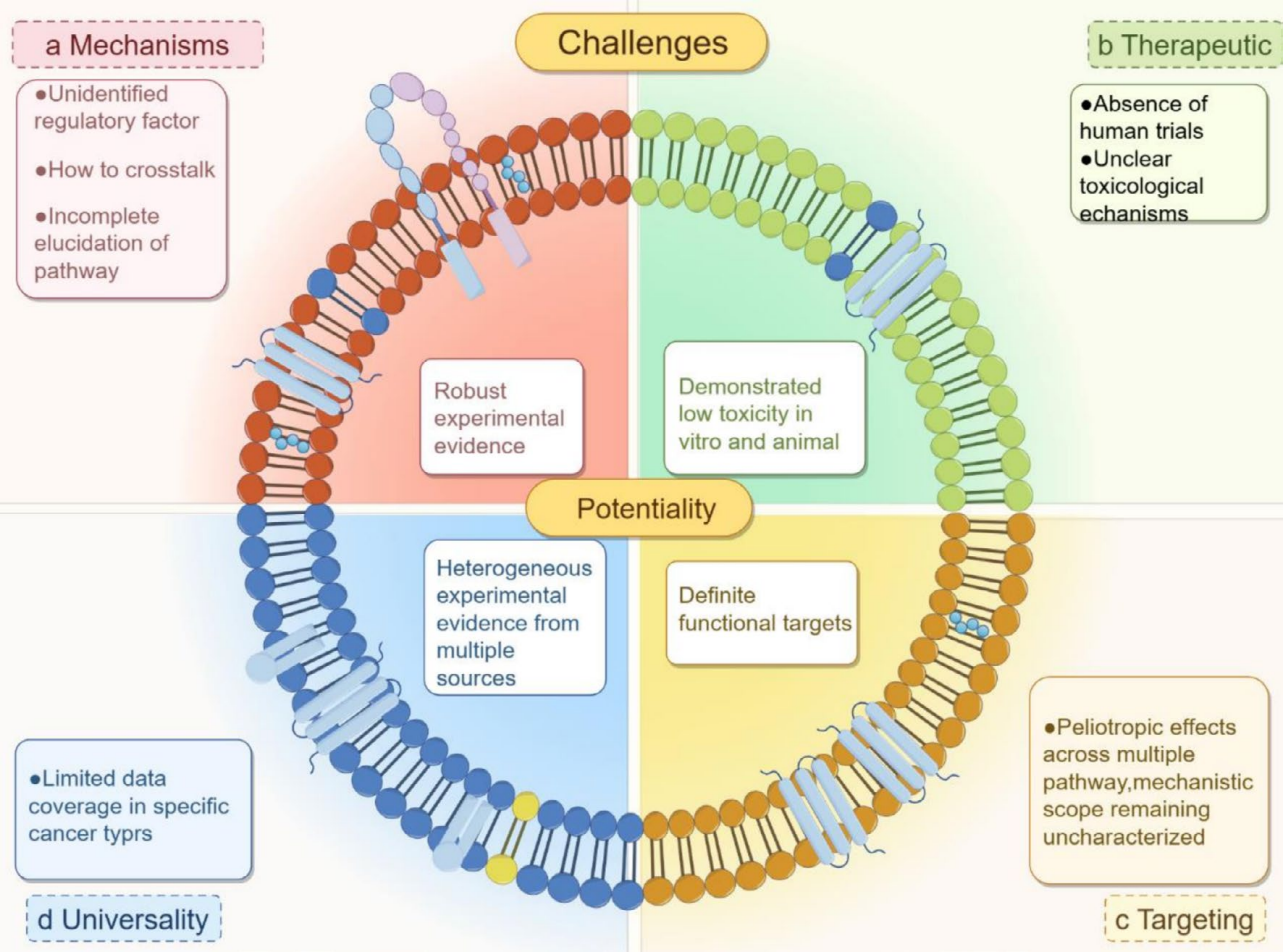
This schematic systematically summarizes current achievements and challenges in the clinical application and development of exosomes, categorized into four dimensions: Mechanisms, Therapeutic Potential, Universality, and Targeting Strategies. In contrast, the exterior represents the associated difficulties and challenges, including issues related to exosomal heterogeneity, delivery efficiency, and off‐target effects.

TEX‐derived ncRNAs reshape the JAK/STAT signaling network through ceRNA mechanisms, where they relieve post‐transcriptional repression of STAT family genes to establish an “RNAs‐miRNAs‐STAT” regulatory axis [16, 32]. For instance, lncRNA HAGLROS sponges miR‐135b‐3p to block its degradation of COL10A1, resulting in COL10A1‐mediated STAT3 phosphorylation and activation of the IL‐10/STAT3 signaling loop that drives breast cancer‐associated M2 polarization [33]. Similarly, lncRNA HEIH sequesters miR‐98‐5p via the ceRNA mechanism, abolishing its direct suppression of STAT3 mRNA and significantly elevating STAT3 protein levels [34]. In the colorectal cancer microenvironment, circPOLQ acts as a miR‐379‐3p sponge to counteract STAT3 inhibition, promoting CD206+ M2 macrophage infiltration—a mechanism functionally complementary to the circATP8A1/miR‐1‐3p/Hippo/STAT6 axis observed in gastric cancer [35, 36]. Notably, certain TEX‐derived ncRNAs (e.g., miR‐210) exhibit dual regulatory modes. First, they inhibit FGFRL1 to relieve JAK2 suppression. Second, they upregulate STAT3 target genes such as Arg1, thereby fostering immunosuppressive microenvironments [22, 23, 24, 25, 37]. The universality of this ceRNA‐mediated network stems from its disruption of the STAT protein synthesis‐degradation equilibrium. This mechanism provides a theoretical foundation for developing targeted therapies based on exosomal RNA components (Figure 3).

### 4.2 Dephosphorylation Inhibition

Recent studies reveal that TEXs establish persistent activation of the JAK/STAT pathway by modulating the phosphorylation‐dephosphorylation equilibrium of STAT proteins, primarily through targeting key molecules responsible for STAT dephosphorylation to sustain nuclear transcriptional activity [38, 39]. For example, miR‐19b‐3p delivered via exosomes to macrophages specifically inhibits protein tyrosine phosphatase receptor type D (PTPRD), blocking its dephosphorylation of STAT3 at the Tyr705 residue. This results in persistent nuclear accumulation of phosphorylated STAT3 and activation of M2 marker genes (e.g., CD206/IL‐10) [40]. A similar mechanism is observed in miR‐510‐mediated direct regulation of STAT3, where stabilization of Tyr705 phosphorylation amplifies JAK2/STAT3 signaling to enhance immunosuppressive functions of TAMs [41]. In gastric cancer microenvironments, SERPINE1 activates the JAK2/STAT3 pathway to promote exosomal packaging of let‐7g‐5p, which suppresses SOCS7 to relieve STAT3 negative feedback, driving STAT3 hyperphosphorylation and M2 polarization [42]. Additionally, miR‐541‐5p targets dual‐specificity phosphatase3 (DUSP3), inhibiting S727 dephosphorylation to maintain JAK2/STAT3 activation. This mechanism increases M2 macrophage infiltration by 2.8‐fold and significantly accelerates tumor angiogenesis in bladder cancer models [43]. Collectively, these findings demonstrate that TEX‐derived ncRNAs induce a “phosphorylation lock” effect by either targeting STAT phosphatases (e.g., PTPRD, DUSP3) or regulating negative feedback suppressors (e.g., SOCS7), with spatiotemporal specificity dictated by selective loading of exosomal RNA components. For instance, circATP8A1 specifically activates M2‐related genes via the miR‐1‐3p/STAT6 axis, while let‐7g‐5p reinforces immunosuppressive microenvironments through the STAT3‐IL‐10 positive feedback loop [36, 43].

### 4.3 SOCS Suppression

Within the TME, TEX‐derived ncRNAs disrupt the negative feedback regulation of the JAK/STAT pathway by targeting Suppressor of Cytokine Signaling (SOCS) family proteins. SOCS members suppress signaling through three mechanisms: (1) binding phosphorylated tyrosine residues on receptors to block STAT recruitment, (2) inhibiting JAK kinase activity, and (3) recruiting E3 ubiquitin ligase complexes for protein degradation [44, 45, 46]. TEX‐derived ncRNAs precisely regulate this network. For instance, miR‐1290 targets SOCS3 mRNA to relieve JAK2/STAT3 inhibition, promoting STAT3 S727 phosphorylation and M2 polarization [47]. Similarly, miR‐106a‐5p suppresses SOCS6 to enhance the activity of the JAK2 V617F mutant [48], while miR‐92b‐5p targets SOCS7 to disrupt STAT3 binding and inhibits DUSP14, thereby sustaining STAT3 Tyr705 phosphorylation and establishing a self‐reinforcing activation loop [49]. These mechanisms collectively shape a pro‐tumorigenic immune microenvironment through hierarchical regulation of SOCS3/6/7 expression and synergistic cross‐activation of STAT3/STAT6 signaling axes.

### 4.4 JAK Activation

Intracellular factors in macrophages, such as Misshapen‐like Kinase 1 (MINK1), Early Growth Response (EGR), and Mechanistic Target of Rapamycin Complex (EGR1), can directly activate the JAK/STAT signaling pathway by modulating JAK kinases, thereby promoting macrophage polarization [50]. For instance, lung adenocarcinoma‐derived miR‐3153 targets ZFP91 to inhibit its E3 ubiquitin ligase activity, reducing ubiquitination‐mediated degradation of MINK1. This sustains JAK1 kinase activity and activates the STAT3 signaling axis, driving M2 polarization [51]. Similarly, circPLEKHM1 enhances OSMR mRNA translation efficiency by stabilizing the PABPC1‐eIF4G complex, which activates the OSMR/JAK2/STAT5b signaling cascade. This synergizes with the IL‐4/STAT6 pathway to amplify TGF‐β secretion [52]. In breast cancer, exosomal miR‐184‐3p binds EGR1 mRNA to relieve its repression of the IL‐4 promoter while simultaneously enhancing Arg1 transcriptional activity through the JNK/c‐Jun axis, establishing a dual metabolic‐immune regulatory network [53]. Additionally, gastric cancer‐derived lncRNA MIR4435‐2HG is delivered via exosomes to interact with the Jagged1 intracellular domain, inhibiting Notch/Hes1 signaling and blocking JAK1/STAT3 negative feedback regulation, thereby promoting IL‐10 secretion [54].

These mechanisms collectively demonstrate that TEXs dynamically regulate the JAK/STAT pathway through a tripartite mode—“kinase activity maintenance‐receptor translation enhancement‐negative regulation suppression”—providing a theoretical basis for developing precision immunotherapies targeting exosomal RNA components.

## 5 PI3K/AKT Signaling Pathway

The PI3K/AKT signaling pathway (Phosphatidylinositol 3‐Kinase/Protein Kinase B signaling pathway)drives macrophage M2 polarization through a multi‐layered regulatory network, with core mechanisms involving metabolic reprogramming, epigenetic regulation, and immune microenvironment remodeling [55]. Upon receptor activation by IL‐4/IL‐13 or similar factors, PI3K catalyzes the generation of phosphatidylinositol (3,4,5)‐trisphosphate (PIP3), recruiting AKT to the cell membrane for dual phosphorylation at Thr308 and Ser473 residues mediated by PDK1/mTORC2, thereby activating AKT [56]. Activated AKT enhances glycolysis (via HK2) and fatty acid oxidation (via CPT1A) through mTORC1 to provide energy for M2 polarization, while phosphorylating and inhibiting GSK3β to relieve its suppression of CREB. This upregulates M2 marker genes such as Arg1 and Ym1 [57, 58, 59]. AKT‐mediated histone acetyltransferase activation further increases chromatin accessibility of M2‐associated genes [60]. Experimental evidence highlights TME‐driven fine‐tuning via TEX‐derived ncRNAs: miR‐21‐5p targets RhoB to relieve PI3K inhibition, activating the SP1/XBP1 axis to promote M2 polarization [61]; miR‐1290 suppresses Akt2 to enhance PD‐L1 expression, driving M2 polarization [62]; and miR‐203a‐3p/miR‐143‐3p alleviates PI3K/AKT pathway suppression by targeting SOCS3, significantly accelerating colorectal cancer liver metastasis [63]. In ovarian cancer models, lncRNA SNHG17 activates PI3K/AKT via the CCL13 chemokine network, upregulating CD163/CD206 expression and accelerating tumor progression. Similarly, oral cancer‐derived lncRNA UCA1 enhances PI3K/AKT activity by sponging miR‐134, inducing M2 polarization and suppressing CD4+ T cell function [64, 65].

### 5.1 PTEN Inhibition

Phosphatase and Tensin Homolog (PTEN), a critical negative regulator of the PI3K/AKT signaling pathway, maintains macrophage polarization homeostasis by catalyzing PIP3 dephosphorylation to PIP2, thereby suppressing AKT activation [66, 67]. Loss‐of‐function mutations or downregulation of PTEN lead to aberrant PI3K/AKT pathway activation, driving macrophage polarization toward the immunosuppressive M2 phenotype. This process is precisely regulated by ncRNA networks [68]. For example, miR‐934 targets PTEN mRNA to inhibit its translation, relieving PI3K suppression and activating the CXCL13‐PI3K/AKT signaling axis to promote M2 polarization and immunosuppressive microenvironment formation [69]. Similarly, miR‐301a‐3p acts as a molecular sponge by competitively binding PTEN mRNA, dually suppressing PTEN protein synthesis and PI3Kγ activity. This significantly enhances pancreatic cancer cell invasiveness and metastasis—effects reversible by the exosome inhibitor GW4869 [70, 71]. Notably, circRNA FARSA recruits E3 ubiquitin ligases via eIF4A3‐mediated circularization to accelerate PTEN degradation. This mechanism functionally complements the loss of lipid phosphatase activity in PTEN secreted mutants (e.g., G129E), synergistically amplifying AKT signaling [72, 73]. In colorectal cancer models, miR‐203a‐3p targets the PTEN/LASP1/GSK‐3β/Snail axis, activating the PI3K/AKT pathway to induce M2 polarization while promoting tumor cell transendothelial migration through cytoskeletal remodeling [74].

### 5.2 KLF‐Dependent Regulation

Krüppel‐like factors (KLFs), core regulatory components of the zinc finger transcription factor family, precisely modulate PI3K/AKT signaling activity through multi‐layered interaction networks. Their functional dysregulation plays a pivotal role in reshaping the tumor immune microenvironment [75]. For instance, KLF4 directly binds to the promoter of the PI3K catalytic subunit p110α to suppress its transcription, significantly reducing PIP3 generation and AKT phosphorylation levels [76]. Experimental evidence demonstrates that the TME dynamically regulates KLF post‐translational modifications and stability via ncRNA networks. In gastric cancer models, lncRNA HCG18 sponges miR‐875‐3p to relieve its suppression of KLF4, promoting SUMOylated KLF4 to synergize with IL‐4 signaling in activating the PI3K/AKT pathway. This drives M2 polarization and accelerates tumor progression—a pathological outcome resembling PI3K/AKT hyperactivation caused by PTEN silencing in multiple myeloma [77, 78, 79]. In non‐small cell lung cancer, lncRNA PCAT6 competitively binds miR‐326 to abolish its inhibition of KLF1, activating PI3K/AKT signaling and inducing EMT. This mechanism intersects with the metabolic‐immune regulatory network formed by KLF2‐mediated cell cycle arrest via p21/p27 during megakaryocyte differentiation [80]. Additionally, miR‐3184‐3p directly suppresses KLF5 mRNA stability, alleviating its transcriptional repression of the PI3K/AKT pathway to promote M2 polarization and enhance tumor immune evasion. This regulatory mode exhibits spatiotemporal heterogeneity, akin to the miR‐25‐3p/PHLPP2 axis‐driven AKT‐mTORC1 activation in hypoxic glioblastoma microenvironments [81, 82, 83].

### 5.3 mTOR Activation and Regulation

mTOR (mechanistic target of rapamycin), a central integrator of the PI3K/AKT signaling pathway, dynamically regulates macrophage polarization through mTOR complex 1 (mTORC1) and mTOR complex 2 (mTORC2) [84]. Its core function lies in coupling metabolic reprogramming with immune phenotype switching: mTORC1 enhances glycolysis (via HK2) and fatty acid oxidation (via CPT1A) to provide energy for M2 polarization while synergizing with HIF‐1α to upregulate anti‐inflammatory factors like IL‐10. Conversely, mTORC2 maintains AKT phosphorylation at Ser473 via positive feedback, amplifying the PI3K/AKT/mTOR signaling cascade [85, 86, 87, 88]. This dynamic regulation is finely orchestrated by ncRNA networks. For example, in glioblastoma microenvironments, miR‐25‐3p targets PHLPP2 to alleviate AKT dephosphorylation inhibition, activating the AKT‐mTORC1 axis to drive M2 polarization [82, 83]. Pancreatic cancer‐derived miR‐4488 suppresses RTN3 to enhance fatty acid oxidation, synergizing with FABP5‐mediated PI3K/AKT/mTOR activation to significantly promote liver metastasis. Similarly, miR‐143‐3p targets PTEN to augment PDK1/mTORC2 activity, fostering a radiotherapy‐resistant M2 phenotype [89, 90]. Meanwhile, LINC00963 degrades Siah1 mRNA to inhibit mTORC1 signaling, collaborating with the PI3K/AKT pathway to regulate mitochondrial translation and sustain M2 homeostasis [91]. Additionally, miR‐99b‐3p inhibits PPP2CA (protein phosphatase 2A catalytic subunit alpha) to activate AKT/mTOR signaling and promote M2 polarization [92]. These findings underscore mTOR's dual role as both a metabolic‐immune nexus and a therapeutic linchpin. Combinatorial strategies using complex‐specific inhibitors (e.g., rapamycin for mTORC1) alongside ncRNA modulation may emerge as pivotal approaches to reverse pathological polarization. Through the content discussed above, it becomes evident that the NF‐κB signaling pathway is not only critically involved in macrophage polarization but also plays a pivotal role in both the PI3K/AKT and JAK/STAT pathways [84, 89]. In fact, several recent cutting‐edge studies have revealed that the NF‐κB pathway exerts complex and subtle influences on TEX‐ncRNAs and the promotion of macrophage polarization [93, 94, 95, 96, 97, 98, 99, 100, 101].

## 6 NF‐κB Signaling Pathway

Recent studies have revealed the dual regulatory role of the Nuclear factor kappa‐B (NF‐κB) signaling pathway in macrophage polarization. Traditionally recognized for its pro‐inflammatory function, the canonical activation pathway is triggered by TNF‐α, IL‐1β, or similar stimuli. This involves IκB kinase (IKK) complex‐mediated phosphorylation and degradation of IκBα, enabling nuclear translocation of NF‐κB dimers (e.g., p65/p50) to activate pro‐inflammatory genes like IL‐6 and TNF‐α, driving macrophage polarization toward the M1 phenotype [102, 103, 104, 105]. However, emerging evidence highlights NF‐κB's critical involvement in M2 polarization through crosstalk with STAT signaling and metabolic reprogramming [93]. For example, acetylation modifications of STAT3 (mediated by circ_0001142 or circ‐001422 via p300 recruitment) enhance nuclear translocation and transcriptional activity of the STAT3/NF‐κB complex, synergistically upregulating M2 markers such as IL‐10 and TGF‐β [94, 95]. Meanwhile, glioma‐derived circNEIL3 is delivered to macrophages via exosomes with hnRNPA2B1 assistance, stabilizing IGF2BP3 to activate NF‐κB and induce CCL5/TGF‐β1 expression, thereby fostering a pro‐tumorigenic M2 microenvironment [96]. Metabolically, NF‐κB suppresses the glycolytic enzyme HK2 to promote oxidative phosphorylation, providing energy for M2 polarization—a process further amplified by miR‐1246 through TERF2IP‐mediated STAT3/NF‐κB axis regulation [97, 98, 99]. Similarly, lncRNA FGD5‐AS1 and miR‐3591‐3p indirectly activate NF‐κB via STAT3 acetylation and CBLB suppression, respectively [100, 101]. These examples underscore the pivotal role of RNA networks in modulating NF‐κB functional switching. Such mechanisms establish positive feedback loops in pathological microenvironments, where tumor cells reshape macrophage NF‐κB activity via exosomal RNA delivery to facilitate immune evasion.

## 7 MAPK/ERK Signaling Pathway

Emerging studies have elucidated that the MAPK/ERK signaling pathway (Mitogen‐Activated Protein Kinase/Extracellular Signal‐Regulated Kinase Signaling Pathway) regulates macrophage M2 polarization through phosphorylation cascades. Under IL‐4/IL‐13 stimulation, this pathway activates ERK via the IRS2‐mediated Ras–Raf–MEK–ERK cascade, inducing metabolic remodeling toward oxidative phosphorylation and fatty acid oxidation to fuel M2 polarization [106, 107]. Phosphorylated ERK translocates to the nucleus, upregulating M2 markers such as Arg1 and CD206 by phosphorylating STAT3 at Ser727, while concurrently activating DUSPs for negative feedback regulation and suppressing the PI3K/AKT pathway to amplify anti‐inflammatory gene expression [108, 109, 110, 111]. For instance, miR‐4655‐5p inhibits MID1 to enhance PP2A phosphatase activity, dampening MAPK‐mediated pro‐inflammatory signaling while activating ERK through an unknown mechanism to promote M2 polarization [112]; lung cancer‐derived exosomal miR‐21 directly activates ERK signaling, with its knockdown reducing ERK activity and significantly decreasing M2 macrophage proportions [113]; miR‐223‐3p binds MEK1 mRNA to boost MEK1 expression, enhancing MAPK/ERK pathway activity and upregulating CD206 to drive M2 phenotypic conversion [114]; miR‐519a‐3p targets DUSP2 mRNA to relieve ERK dephosphorylation inhibition, sustaining MAPK/ERK activation and promoting M2 macrophage‐mediated angiogenesis in gastric cancer liver metastasis [115]; circPVT1 sponges miR‐124‐3p to upregulate EZH2, which suppresses PAK1 mRNA degradation via H3K27 trimethylation, thereby activating the MAPK pathway [116]; lncRNA SLC16A1‐AS1 binds HNRNPA1 to stabilize SLC16A1 mRNA, enhancing lactate transport to activate the c‐Raf/ERK axis and ultimately inducing M2 polarization while accelerating hepatocellular carcinoma progression [117].

## 8 HIF‐1α/PKM2 Signaling Axis

Hypoxia‐inducible factor (HIF), a central regulator of cellular metabolism and hypoxia adaptation, plays a pivotal role in tumor metabolic reprogramming and macrophage M2 polarization, with HIF‐1α being the key mediator [118]. Under hypoxic conditions, HIF‐1α is stabilized by evading ubiquitin‐proteasomal degradation and enhances glycolytic capacity via upregulation of pyruvate kinase M2 (PKM2), leading to lactate accumulation and elevated intracellular calcium levels that drive macrophage polarization toward the M2 phenotype [119, 120, 121, 122]. Simultaneously, HIF‐1α reinforces the immunosuppressive and tissue‐repair functions of macrophages by activating M2 hallmark cytokines such as IL‐10 and TGF‐β [123, 124]. Studies reveal that multiple RNAs regulate PKM2 and glycolysis through HIF‐1α‐dependent mechanisms. For example, circSHKBP1 sponges miR‐1294 to relieve its suppression of PKM2, promoting glycolytic activity and M2 polarization of TME [125]; lncRNA ElNF1‐AS1 upregulates PKM2 by targeting miR‐4644, synergizing with HIF‐1α to enhance glycolysis and pro‐tumorigenic phenotypes in gastric cancer‐associated macrophages [126]; while lncRNA ZFPM2‐AS1 competitively binds miR‐18b‐5p to counteract PKM2 inhibition, fostering HIF‐1α‐mediated M2 polarization and tumor immune evasion [127]. Additionally, miR‐452‐5p indirectly modulates the HIF‐1α/PKM2 axis by suppressing TIMP3, which inhibits MMP9 expression and remodels the metabolic niche to induce M2 polarization in hepatocellular carcinoma microenvironments [128].

## 9 Wnt/β‐Catenin Signaling Pathway

The Wnt/β‐catenin signaling pathway, a critical network regulating embryonic development, tissue homeostasis, and tumorigenesis, plays a central role in macrophage M2 polarization by controlling β‐catenin stability and nuclear translocation [129]. Upon activation, Wnt ligands bind to Frizzled receptors and LRP5/6 co‐receptors, triggering Disheveled protein recruitment and inhibiting the destruction complex (composed of APC, Axin, GSK3β, and CK1), thereby preventing β‐catenin phosphorylation and subsequent ubiquitin‐proteasomal degradation [130, 131]. Stabilized β‐catenin accumulates in the cytoplasm and translocates to the nucleus, where it binds TCF/LEF transcription factors to activate target genes such as c‐Myc. These genes regulate immune factors (e.g., IL‐10) and metabolic pathways (e.g., glycolysis, glutamine metabolism), providing energy and an immunosuppressive microenvironment for M2 polarization [132, 133, 134]. For example, miR‐138‐5p targets the histone demethylase KDM6B to block its β‐catenin degradation complex activity, activating the β‐catenin/c‐Myc axis and driving M2 polarization in breast cancer microenvironments [135]; circRHCG is delivered via exosomes to macrophages, where it binds the RNA‐binding protein FUS to stabilize BTRC mRNA, enhancing TFEB ubiquitination and degradation. This relieves TFEB‐mediated inhibition of the β‐catenin‐TCF/LEF1 complex, ultimately promoting M2 phenotypic conversion through c‐Myc upregulation [136]. Additionally, miR‐21 inhibits the E3 ubiquitin ligase PELI1 to reduce Foxp1 degradation, amplifying β‐catenin transcriptional activity to foster M2 polarization [137], while miR‐200b‐3p targets ZEB1 to promote PPARγ dephosphorylation and nuclear translocation. This synergizes with β‐catenin to regulate lipid synthesis genes (e.g., FAS), forming a positive feedback loop between metabolic reprogramming and the Wnt pathway to sustain the immunosuppressive functions of M2 macrophages [138]. The conventional signaling pathways discussed above provide a relatively comprehensive overview of the relationship between TEX‐ncRNAs and macrophage polarization. However, some studies have revealed mechanisms that do not involve specific signaling pathways but rather directly affect the immune environment and macrophages through metabolic regulation or core molecular interactions [139, 140, 141].

## 10 Other Regulatory Mechanisms

TEX‐derived ncRNAs can promote M2 polarization by dynamically interacting with specific proteins or RNA‐binding proteins beyond classical signaling pathways, influencing the stability and functionality of key factors. These mechanisms primarily involve metabolic reprogramming of target macrophages and stabilization of IL‐4 mRNAs, which are crucial regulatory hubs in this process (Table 1).

**TABLE 1:** Name of TEX‐derived ncRNAs, targeted signaling pathways, tumor types, experimental models, and key experimental findings.

Signaling pathway	Non‐coding RNA	Experimental models	Key experimental findings	Cancer type	References
JAK/STAT	lncRNA NEAT1	Astrocytoma xenograft mouse model; macrophage‐exosome co‐culture system	High NEAT1 expression remodels the immunosuppressive microenvironment and accelerates tumor progression	Glioma	[26]
	lncRNA HAGLROS	Breast cancer metastasis mouse model; macrophage‐exosome co‐culture system	Exosome treatment increased p‐STAT3 levels by 1.5‐ to 2‐fold	Breast cancer	[33]
	lncRNA HEIH	Hepatocellular carcinoma xenograft model; macrophage‐exosome co‐culture system	HEIH knockdown significantly suppressed tumor progression and reduced gemcitabine resistance	Liver cancer	[34]
	miR‐210	Macrophage‐exosome co‐culture system	miR‐210 overexpression enhanced gemcitabine resistance by 2–3‐fold	Pancreatic cancer	[37]
	circPOLQ	Colorectal cancer liver metastasis mouse model; macrophage‐exosome co‐culture system	High circPOLQ expression in plasma exosomes correlates with poor survival	Colorectal cancer	[35]
	circATP8A1	Gastric cancer xenograft model; macrophage‐exosome co‐culture system	circATP8A1 overexpression significantly reduced mouse survival	Gastric cancer	[36]
	miR‐19b‐3p	Lung metastasis mouse model	Inhibition of miR‐19b‐3p or LINC00273 significantly reduced metastatic foci	Lung cancer	[40]
	miR‐510	Macrophage‐pancreatic cancer cell co‐culture system	miR‐510 overexpression increased tumor cell invasion/migration by 40%–60%	Pancreatic cancer	[41]
	let‐7 g‐5p	Gastric cancer xenograft model	let‐7 g‐5p knockdown suppressed tumor growth and reduced M2 macrophage infiltration	Gastric cancer	[42]
	miR‐541‐5p	Gastric cancer xenograft model; macrophage‐gastric cancer cell co‐culture system	miR‐541‐5p overexpression significantly decreased mouse survival	Gastric cancer	[43]
	miR‐1290	Lung adenocarcinoma mouse model	miR‐1290 inhibition reduced lung metastasis by 50%	Lung cancer	[47]
	miR‐106a‐5p	Colorectal cancer liver metastasis mouse model	miR‐106a‐5p overexpression increased liver metastatic foci by 3‐fold	Colorectal cancer	[48]
	miR‐92b‐5p	Gastric cancer xenograft model; macrophage‐exosome co‐culture system	PLXNC1 overexpression increased M2 macrophage infiltration by 2.3‐fold	Gastric cancer	[49]
	miR‐3153	Orthotopic lung cancer mouse model	miR‐3153 inhibition reduced lung metastasis by 70%	Lung cancer	[51]
	circPLEKHM1	Lung cancer bone metastasis mouse model	circPLEKHM1 knockdown reduced bone metastasis by 60%	Lung cancer	[52]
	miR‐184‐3p	Breast cancer lung metastasis mouse model; macrophage‐exosome co‐culture system	hnRNPA2B knockdown reduced IL‐10 secretion by 60% and lung metastasis by 65%	Breast cancer	[53]
	lncRNA MIR4435‐2HG	Gastric cancer xenograft model; macrophage‐exosome co‐culture system	MIR4435‐2HG overexpression increased STAT6 phosphorylation by 2‐fold	Gastric cancer	[54]
PI3K/AKT	miR‐21‐5p	Macrophage‐exosome co‐culture system; hepatocellular carcinoma xenograft model	miR‐21‐5p overexpression increased AKT phosphorylation by 2‐fold; knockdown reduced tumor volume by 40% and M2 infiltration by 50%	Liver cancer	[61]
	miR‐1290	Exosome co‐culture with CD8+ T cells/macrophages; liver cancer xenograft model	miR‐1290 inhibition reduced tumor volume by 50% and increased CD8+ T cell infiltration by 1.8‐fold	Liver cancer	[62]
	miR‐203a‐3p；miR‐143‐3p	Colorectal cancer liver metastasis mouse model; macrophage‐exosome co‐culture system	Exosome levels correlated with liver metastasis risk (HR = 2.3); PTEN protein levels decreased by 70%	Colorectal cancer	[63]
	lncRNA SNHG17	Ovarian cancer peritoneal metastasis mouse model	SNHG17 knockout reduced peritoneal metastasis by 60% and extended survival by 30%	Ovarian cancer	[64]
	lncRNA UCA1	Oral cancer xenograft model	UCA1 inhibition reduced tumor volume by 45% and decreased T cell infiltration by 50%	Oral cancer	[65]
	miR‐934	Colorectal cancer liver metastasis mouse model	miR‐934 overexpression increased liver metastasis by 3‐fold	Colorectal cancer	[69]
	miR‐301a‐3p	Esophageal cancer xenograft model; pancreatic cancer lung metastasis mouse model	miR‐301a‐3p inhibition significantly reduced lung metastasis	Esophageal cancer、Pancreatic cancer	[70, 71]
	circRNA FARSA	Orthotopic lung cancer mouse model	circFARSA knockout reduced metastasis by 65%	Lung cancer	[72]
	miR‐203a‐3p	Colorectal cancer liver metastasis mouse model	miR‐203a‐3p inhibition reduced liver metastasis by 40%	Colorectal cancer	[74]
	lncRNA HCG18	Gastric cancer xenograft model; macrophage‐gastric cancer cell co‐culture system	HCG18 knockdown reduced tumor volume by 40% and M2 macrophage proportion by 50%	Gastric cancer	[78, 79]
	lncRNA PCAT6	Orthotopic lung cancer mouse model; macrophage‐lung cancer cell co‐culture system	PCAT6 knockdown reduced tumor volume by 45% and KLF1 expression by 60%	Lung cancer	[80]
	miR‐3184‐3p	Macrophage‐exosome co‐culture system; glioma orthotopic mouse model	miR‐3184‐3p overexpression increased STAT3 phosphorylation by 2.5‐fold	Glioma	[81]
	miR‐25‐3p	Glioma orthotopic mouse model; macrophage‐exosome co‐culture system	miR‐25‐3p overexpression increased CD206+ cells by 70%; knockdown reduced tumor volume by 55%	Glioma	[82, 83]
	miR‐4488	Liver metastasis mouse model	miR‐4488 inhibition reduced liver metastasis by 60%	Pancreatic cancer	[89]
	miR‐143‐3p	Esophageal cancer radiotherapy‐resistant mouse model; macrophage‐exosome co‐culture system	miR‐143‐3p overexpression decreased PTEN protein by 50% and increased tumor recurrence by 2‐fold	Esophageal cancer	[90]
	lncRNA LINC00963	Lung cancer metastasis mouse model	LINC00963 knockdown significantly reduced lung metastasis	Lung cancer	[91]
	miR‐99b‐3p	Chemotherapy‐sensitive breast cancer mouse model	miR‐99b‐3p inhibition significantly reduced post‐chemotherapy tumor volume	Breast cancer	[92]
NF‐κB	circNEIL3	Glioma xenograft mouse model	circNEIL3 knockdown reduced tumor volume by 55% and macrophage infiltration by 60%	Glioma	[96]
	miR‐1246	Glioma metastatic mouse model	miR‐1246 inhibition significantly reduced tumor volume and metastasis	Glioma	[99]
	circ_0001142	Breast cancer xenograft model	circ_0001142 knockdown decreased proliferation by 50% and migration by 60%	Breast cancer	[94]
	miR‐3591‐3p	Glioblastoma intracranial xenograft mouse model	miR‐3591‐3p knockdown reduced tumor volume by 55% and extended survival by 30%	Glioma	[100]
	circ‐001422	Glioma orthotopic mouse model	circ‐001422 knockdown decreased invasiveness and extended survival	Glioma	[95]
	lncRNA FGD5‐AS1	Pancreatic cancer liver metastasis mouse model	FGD5‐AS1 inhibition reduced liver metastasis and enhanced chemotherapy sensitivity	Pancreatic cancer	[101]
MAPK/ERK	miR‐4655‐5p	Lung cancer metastasis mouse model	miR‐4655‐5p inhibition reduced lung metastasis and M2 macrophage infiltration	Lung cancer	[112]
	miR‐21	Lung cancer brain metastasis mouse model	miR‐21 inhibition significantly reduced brain tumor volume	Lung cancer	[113]
	miR‐223‐3p	Sleep deprivation‐induced colorectal cancer xenograft mouse model	CD206+ macrophages increased by 50%, promoting tumor proliferation and migration	Colorectal cancer	[114]
	miR‐519a‐3p	Gastric cancer liver metastasis mouse model	miR‐519a‐3p inhibition reduced liver metastasis by 60%; overexpression increased aortic ring sprouting by 80%	Lung cancer	[115]
	circPVT1	Orthotopic lung cancer mouse model	circPVT1 knockdown extended survival	Liver cancer	[116]
	lngRNA SLC16A1‐AS1	Hepatocellular carcinoma xenograft model; macrophage‐exosome co‐culture system	SLC16A1‐AS1 knockdown reduced tumor volume by 50% and metastasis by 60%; overexpression increased HIF‐1α (1.5‐fold) and 14C‐lactate uptake (70%)	Liver cancer	[117]
HIF‐1α/PKM2	circSHKBP1	Lung cancer xenograft model; macrophage‐lung cancer cell co‐culture system	circSHKBP1 overexpression increased lactate production by 40%; knockdown reduced tumor volume by 40% and M2 macrophages	Lung cancer	[125]
	lncRNA ElNF1‐AS1	Gastric cancer liver metastasis mouse model; macrophage‐gastric cancer cell co‐culture system	ELFN1‐AS1 inhibition reduced liver metastasis by 60%; overexpression increased IL‐10 secretion by 1.8‐fold	Gastric cancer	[126]
	lncRNA ZFPM2‐AS1	Hepatocellular carcinoma orthotopic mouse model; macrophage‐liver cancer cell co‐culture system	ZFPM2‐AS1 overexpression increased glucose uptake and CD206+ macrophage proportion	Liver cancer	[127]
	miR‐452‐5p	Liver cancer metastasis mouse model	miR‐452‐5p overexpression significantly upregulated Arg‐1	Liver cancer	[128]
Wnt/β‐catenin	miR‐138‐5p	Breast cancer lung metastasis mouse model; macrophage‐breast cancer cell co‐culture system	miR‐138‐5p overexpression decreased KDM6B by 60% and increased CD163+ macrophages by 70%	Breast cancer	[135]
	circRHCG	Triple‐negative breast cancer xenograft model; macrophage‐breast cancer cell co‐culture system	circRHCG overexpression increased TFEB ubiquitination by 1.5‐fold and IL‐10 secretion	Breast cancer	[136]
	miR‐21	Triple‐negative breast cancer metastasis mouse model; macrophage‐breast cancer cell co‐culture system	miR‐21 overexpression reduced Foxp1 degradation and increased CD206+ macrophages by 50%	Breast cancer	[137]
	miR‐200b‐3p	Hepatocellular carcinoma orthotopic model; macrophage‐exosome co‐culture system	miR‐200b‐3p overexpression significantly increased TGF‐β secretion	Liver cancer	[138]
Others	circGLIS3	Gastric cancer xenograft model	circGLIS3 overexpression increased glycolysis by 60% and migration by 70%	Gastric cancer	[139]
	lncRNA HMMR‐AS1	Liver cancer metastasis mouse model; macrophage‐exosome co‐culture system	HMMR‐AS1 overexpression increased CD206+ macrophages by 60%	Liver cancer	[141]
	mRNA RNF157	Prostate cancer bone metastasis mouse model; macrophage‐exosome co‐culture system	RNF157 overexpression increased HDAC1 ubiquitination by 70% and upregulated Arg‐1	Prostate cancer	[142]

### 10.1 Glycolysis‐Related Metabolic Reprogramming

Exosomal mRNAs can not only function through specific signaling pathways but also directly regulate nucleic acids related to metabolic reprogramming enzymes in target macrophages, thereby influencing metabolic reprogramming. This process enhances energy production in target cells to meet the metabolic demands of M2 polarization, ultimately promoting the M2 phenotype. For example, ovarian cancer‐derived exosomal circGLIS3 drives M2 polarization through dual mechanisms: it sponges miR‐1343‐3p to relieve its suppression of PGK1 (phosphoglycerate kinase 1), enhancing glycolytic metabolism, while directly binding vimentin to inhibit its phosphorylation, thereby blocking vimentin‐STAT3 complex dissociation and facilitating STAT3 nuclear translocation [139]. Notably, certain exosomal components exert effects via epigenetic reprogramming: RNF157 mRNA binds the catalytic domain of HDAC1 (histone deacetylase 1) to promote its ubiquitination‐mediated degradation, elevating histone H3K27 acetylation levels and relieving transcriptional repression of the IL‐10 locus [142].

### 10.2 IL‐4 mRNA Stabilization

Additionally, specific exosome‐derived lncRNAs can bind to IL‐4‐related mRNAs, thereby stabilizing molecular structures or activating their promoters, which facilitates the formation of an anti‐inflammatory microenvironment and promotes M2 polarization. For instance, circRNA hsa_circ_0074854 binds the RRM3 domain of the ELAVL1 protein to stabilize IL‐4 mRNA and suppress TNF‐α translation efficiency, creating an anti‐inflammatory microenvironment [140]. In hepatocellular carcinoma models, lncRNA HMMR‐AS1 sponges miR‐147a to prevent ARID3A degradation via targeted decay. ARID3A then directly binds the IL‐4 promoter to amplify its transcriptional activity, thereby promoting M2 macrophage polarization [141].

This mechanistic diversity underscores the need for tailored therapeutic strategies targeting ncRNAs based on their functional tiers—whether metabolic enzymes (e.g., PGK1), epigenetic regulators (e.g., HDAC1), or cytoskeletal proteins (e.g., vimentin)—to disrupt the multi‐tiered regulatory logic of M2 polarization.

## 11 Discussion

TEXs have emerged as a central mechanism in regulating the immunosuppressive TME by delivering non‐coding RNAs (ncRNAs) to reshape macrophage polarization states. This review systematically deciphers the cascading regulatory networks through which exosomal ncRNAs modulate M2 polarization via signaling pathways such as JAK/STAT, PI3K/AKT, and NF‐κB. These mechanisms span multiple dimensions, including ceRNA interactions, epigenetic reprogramming, and metabolic dysregulation. This review highlights six signaling pathways essential in M2 polarization and tumor progression: JAK/STAT promotes metastasis and therapy resistance via SOCS/Arg1/PD‐L1 [22, 23, 24, 25]; PI3K/AKT drives early tumorigenesis through PTEN/mTOR/HIF‐1α‐mediated metabolic reprogramming [55, 56, 57, 58, 59, 60]; NF‐κB supports tumor promotion and metastatic niches by suppressing M1 genes and synergizing with STAT3/AKT [102, 103, 104, 105]; MAPK/ERK influences all cancer stages by regulating transcription factors and inflammatory responses [106, 107]; HIF‐1α/PKM2 and Wnt/β‐catenin aid hypoxia adaptation and recurrence via glycolytic reprogramming and M2 marker expression [118, 129]. These pathways have been extensively validated through robust experimental evidence, establishing their critical functions during macrophage polarization and positioning them as major research focuses in this field in recent years [17, 18, 19, 22]. Furthermore, these signaling cascades are closely linked to clinical applications, as they directly interface with the mechanisms of action of immunosuppressive agents such as PD‐L1 inhibitors [10, 11, 12, 13]. Most importantly, they represent the most essential and central downstream hubs of TEX‐ncRNAs‐mediated regulation—direct perturbation of these pathways profoundly disrupts the ability of TEX‐ncRNAs to influence polarization [20]. Current studies highlight the pan‐cancer applicability and target specificity of this mechanism—targeted inhibition of key exosomal ncRNAs in preclinical models significantly reverses M2 polarization without observable toxicity, while their chemical modifiability offers an engineerable window for drug development. However, critical challenges persist: (1) The transmembrane delivery mechanisms of TEX‐derived ncRNAs (e.g., Rab27a/SNARE complexes vs. non‐classical secretion pathways) and their spatiotemporal activation patterns remain elusive; (2) The hierarchical regulation of dynamic signaling crosstalk (e.g., STAT3‐NF‐κB positive feedback loops) in M2 polarization requires systematic elucidation; (3) Existing evidence is largely confined to common cancers (e.g., gastric/lung cancers), with limited validation in rare malignancies like nasopharyngeal carcinoma; (4) Clinical translation faces hurdles such as exosomal heterogeneity, off‐target effects, and delivery inefficiency. To address these challenges and further advance the development of exosome‐based cancer therapies, it is crucial to focus on exploiting their potential in targeted treatment. To improve targeting efficiency, engineered exosomes could be decorated with ligands specific to receptors highly expressed on TAMs or other components of the TME, such as folate receptor beta or CD206. Given that combination precision therapy has become a major direction in cancer treatment, combining exosomal ncRNA inhibitors with immune checkpoint blockers has shown synergistic effects in reversing immunosuppression and enhancing antitumor immunity. This represents a promising combinatorial strategy worthy of further exploration. Multi‐omics approaches, particularly single‐cell RNA sequencing coupled with exosomal RNA profiling, can decipher the heterogeneity of both tumor cells and TAMs. This will help identify specific exosomal ncRNA signatures associated with particular macrophage subtypes and functional states, paving the way for more precise targeting strategies. Future research should prioritize multi‐omics integration to resolve the spatial specificity of ncRNA‐target interactions and develop precision‐engineered exosome‐based strategies (e.g., CRISPR/dCas13‐mediated ncRNA editing). Thus, decoding the molecular logic of exosomal ncRNA‐mediated M2 polarization will not only refine the theoretical framework of tumor immune editing but also potentially unlock new avenues to overcome immunotherapy resistance.

## Author Contributions

**Yifan Bian:** writing – original draft, writing – review and editing. **Jiarui Cao:** writing – review and editing. **Jilei Li:** writing – review and editing. **Sizhe Wang:** writing – review and editing. **Chunzheng Ma:** supervision.

## Funding

The author(s) declare that financial support was received for the research, authorship, and/or publication of this article. This work is supported by the Henan Province Administration of Traditional Chinese Medicine (Nos. 2022ZY1089, 2024ZY1013), Leading Talents of Henan Province (No. (2021)‐8), and Henan Provincial Hospital of Traditional Chinese Medicine Doctoral Fund (No. 2024BSJJ10).

## Disclosure

Declaration of generative AI and AI‐assisted technologies in the writing process: The author(s) declare that no Generative AI was used in the creation of this manuscript.

## Conflicts of Interest

The authors declare no conflicts of interest.

## Data Availability

The authors have nothing to report.
